# Primary Cilia Dysfunction in Neurodevelopmental Disorders beyond Ciliopathies

**DOI:** 10.3390/jdb10040054

**Published:** 2022-12-13

**Authors:** Vasiliki Karalis, Kathleen E. Donovan, Mustafa Sahin

**Affiliations:** 1 The Rosamund Stone Zander Translational Neuroscience Center, Department of Neurology Boston Children’s Hospital, Harvard Medical School, Boston, MA 02115, USA; 2 FM Kirby Neurobiology Center, Boston Children’s Hospital, Boston, MA 02115, USA

**Keywords:** primary cilia, ciliopathies, neurodevelopmental disorders, signaling

## Abstract

Primary cilia are specialized, microtubule-based structures projecting from the surface of most mammalian cells. These organelles are thought to primarily act as signaling hubs and sensors, receiving and integrating extracellular cues. Several important signaling pathways are regulated through the primary cilium including Sonic Hedgehog (Shh) and Wnt signaling. Therefore, it is no surprise that mutated genes encoding defective proteins that affect primary cilia function or structure are responsible for a group of disorders collectively termed ciliopathies. The severe neurologic abnormalities observed in several ciliopathies have prompted examination of primary cilia structure and function in other brain disorders. Recently, neuronal primary cilia defects were observed in monogenic neurodevelopmental disorders that were not traditionally considered ciliopathies. The molecular mechanisms of how these genetic mutations cause primary cilia defects and how these defects contribute to the neurologic manifestations of these disorders remain poorly understood. In this review we will discuss monogenic neurodevelopmental disorders that exhibit cilia deficits and summarize findings from studies exploring the role of primary cilia in the brain to shed light into how these deficits could contribute to neurologic abnormalities.

## 1. Introduction 

The first observation of cilia was reported in 1674–1675, when Antoni van Leeuwenhoek was studying pond protozoa and noticed small hairlike structures that he described as “incredibly thin feet, which moved very nimbly” [1]. Later, these structures were identified as cilia, a highly conserved organelle that is currently classified into two groups: motile and non-motile cilia, with the latter group being much less understood. Both motile and non-motile cilia contain an axoneme, a structure made of nine peripheral microtubule doublets. In motile cilia, there is a central pair of microtubules (referred to as 9 + 2 arrangement), which is lacking in non-motile cilia (referred to as 9 + 0 arrangement). However, not all non-motile cilia conform to this 9 + 0 arrangement and among the ones that do, some exhibit motility, suggesting that there might be structural variability within these groups [2,3,4]. Non-motile cilia, today called primary cilia, are present in almost all types of cells in the body and are thought to serve mainly as ‘the cell’s antennae’, receiving and integrating extracellular chemical and mechanical signals. 

**Figure 1:**
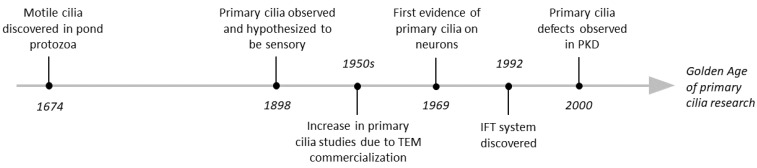
Key events in primary cilia research history. Schematic illustration of the timeline of the major discoveries in the field of primary cilia. (TEM) Transmission electron microscopy, (IFT) Intraflagellar transport, (PKD) Polycystic kidney disease.

Although primary cilia were observed as early as 1898 [5], their study was delayed for almost 50 years due to a lack of tools [4]. Only after the advent of transmission electron microscopy (TEM) and its subsequent commercialization did the primary cilia field gain minor traction in the 1950s and 60s [6,7,8]. For most of the 20th century, primary cilia were considered “rudimentary” or “vestigial” organelles [9,10]. However, in 1992, the ciliary intraflagellar transport (IFT) system was discovered [11] and associated with polycystic kidney disease [12], indicating that these organelles had a more important role than previously thought. These discoveries catalyzed our current ‘Golden Age’ of primary cilia research, in which much has been elucidated about primary cilia’s biological relevance (Figure 1). 

The primary cilium’s function is closely related to the cell cycle. Specifically, primary cilia formation occurs during the G_1_/G_0_ phase, followed by resorption upon cell cycle re-entry [13]. Ciliogenesis is initiated upon formation of the basal body from the centrosome (the mother and daughter centriole pair). The basal body docks at the apical plasma membrane, and microtubules nucleate to begin axoneme formation [14], with the axoneme projecting outward beneath the plasma membrane [15]. The region where the basal body and axoneme meet is called the transition zone, and it is required for cilium compartmentalization. In the transition zone, Y-shaped protein link the axoneme to the plasma membrane, restricting the movement of proteins and lipids into and out of the ciliary compartment [16]. To elongate the cilium, proteins are selectively imported and transported to the ciliary tip via anterograde transport by the IFT system. Anterograde and retrograde ciliary transport are regulated by distinct IFT complex/motor protein pairs: IFT-B with homotrimeric Kinesin-2, and IFT-A with cytoplasmic dynein 2, respectively [15,17]. Proteins which enter the cilium via anterograde transport can be removed through retrograde transport or secreted within ciliary ectosomes to the extracellular space [18,19].

The primary cilium actively imports receptors and signaling molecules [20,21] making it uniquely suited to sense and integrate signals. Several signaling pathways are coordinated through the primary cilium, including Sonic Hedgehog (Shh), G-protein-coupled receptors (GPCR), platelet-derived growth factor receptor α (PDGFα), fibroblast growth factor (FGF), transforming growth factor β (TGF-β), Wnt, Hippo, Notch, and mechanistic target of rapamycin (mTOR) signaling. These pathways have been extensively reviewed elsewhere [21,22].

The first evidence for the presence of primary cilia on neurons was reported in 1969 [23]. However, their contribution to brain development and function was not investigated until the early 21st century [24,25,26] (Figure 1). Today it is known that the majority of mature neurons and astrocytes in the central nervous system (CNS) have a primary cilium, but so far primary cilia have not been detected on mature oligodendrocytes or microglia [27]. Mutations in genes that affect primary cilia have major neurologic consequences and are currently an active field of research. Interestingly, recent studies identified impaired neuronal primary cilia in monogenic neurodevelopmental disorders in which the disease-causing mutations do not have clear links to the primary cilium. In this review we discuss (1) monogenic neurodevelopmental disorders that were shown to exhibit impaired ciliation and (2) evidence that could shed light on the mechanisms via which defective cilia could contribute to these disorders’ neurologic manifestations. 

## 2. Primary Cilia Deficits in CNS Disorders: Ciliopathies and Beyond

Primary cilia host some of the most important signaling pathways for proper brain development and function. Several of these signaling pathways function exclusively through cilia, making these organelles vital cellular compartments [21,22]. Therefore, it is not surprising that defects in cilia underlie several disorders collectively termed “ciliopathies” [28].

Ciliopathies are a group of inherited genetic disorders caused by mutations in genes encoding proteins essential for the structure and function of cilia [29]. Since cilia are components of nearly all human cells, pathological manifestations of ciliopathies are multisystemic, and include retinopathy, obesity, diabetes, skeletal malformations, and hepatic disease [29,30]. Interestingly, certain ciliopathies specifically affect only a subset of organs such as polycystic kidney disease (PKD), which is characterized by multiple cysts in the kidney and liver [29,30]. 

The importance of cilia function in the brain is underscored by the presence of a wide range of neurologic manifestations in ciliopathies such as Joubert and Bardet–Biedl syndromes. These disorders exhibit brain malformations, ataxia, and cognitive deficits [31,32]. In addition to the role of cilia dysfunction in ciliopathies, cilia deficits have been observed in a broad range of CNS disorders suggesting that these organelles might contribute to the pathology of disorders beyond ciliopathies [33,34,35]. Specifically, it has been proposed that abnormal primary cilia function may be involved in neuropsychiatric conditions, such as schizophrenia and bipolar disorder [33]. In addition, recent reports implicate cilia in neurodegenerative disorders such as Alzheimer’s and Parkinson’s disease [34,35]. These data emphasize the importance of primary cilia in the proper development and function of the brain across the lifespan. 

While cilia dysfunction plays a clear role in the development of neurological manifestations in ciliopathies, there is increasing evidence that other monogenetic neurodevelopmental syndromes indirectly impinge on primary cilia function. This has provided the intriguing hypothesis that ciliary dysfunction may be an integral part of the pathological mechanisms underlying these disorders. Therefore, these disorders provide a further framework to investigate (1) novel key players in the molecular mechanisms that regulate primary cilia structure and function and (2) the roles of primary cilia in normal and diseased brain. 

## 3. Primary Cilia Defects in Monogenic Neurodevelopmental Disorders 

**Figure 2:**
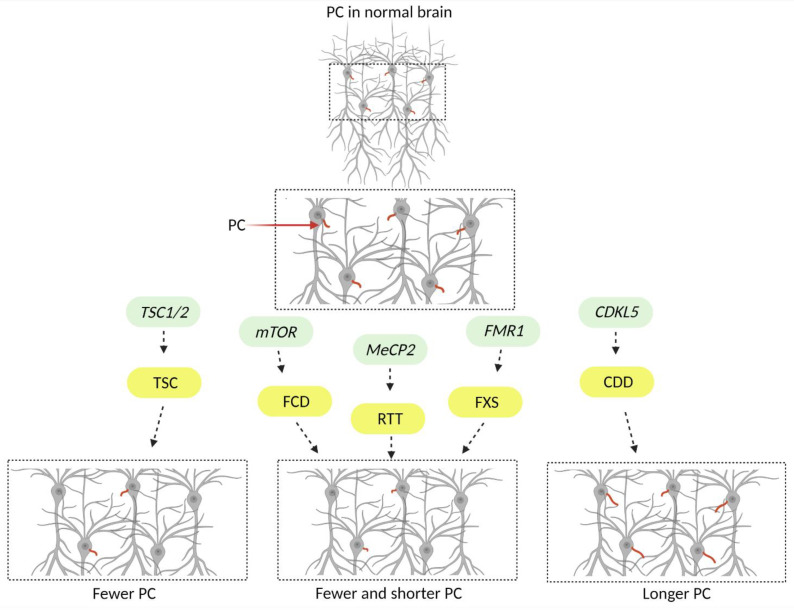
Primary cilia phenotypes in monogenic neurodevelopmental disorders. Mutations in genes (denoted in green) underly a group of neurodevelopmental disorders (denoted in yellow) that exhibit severe neurologic and neuropsychiatric manifestations including cortical malformation, epilepsy, ASD, and ID. Some of the neurological phenotypes are unique to certain disorders while others are shared (see Table 1). Recently several studies discovered that these disorders exhibit neuronal primary cilia (colored red in the illustration) deficits raising the question of whether these organelles contribute to the neurologic manifestations that patients exhibit. (TSC) Tuberous sclerosis complex, (FCD) Focal cortical dysplasia, (RTT) Rett syndrome, (FXS) Fragile-X syndrome, (CDD) CDKL5 deficiency disorder, (ASD) Autism spectrum disorder, (ID) Intellectual disabilities. Created with BioRender.com (www.biorender.com, accessed on 2 December 2022).

Due to the significant role of neuronal primary cilia in the brain, studies have investigated whether and how these organelles are impacted in developmental disorders with severe neuropsychiatric manifestations. A group of monogenic neurodevelopmental disorders that exhibit several common neurologic manifestations and were found to have defects in neuronal primary cilia are discussed below. Figure 2 and Table 1 summarize the findings of the studies, focusing on neuronal primary cilia. 

### 3.1. Fragile X Syndrome 

Fragile X syndrome (FXS) is caused by loss of function of the fragile X messenger ribonucleoprotein (FMRP), which is typically caused by epigenetic silencing of the *FMR1* gene due to an expanded CGG repeat in the promoter [50]. FMRP is a widely expressed RNA-binding protein and a translational repressor that has been linked to proper development and maintenance of neuronal synapses. Patients with FXS present with a range of neurological symptoms, including intellectual disability, autism spectrum disorder (ASD), and seizures [36,38]. 

A recent study by Lee et al. showed that *Fmr1*-KO mice exhibit reduced primary cilia number and length, within mature granule neurons of the dentate gyrus (DG) after postnatal day 14 (Figure 2, Table 1). Interestingly, these primary cilia abnormalities were not observed in other hippocampal regions [37]. In addition, when the authors examined the two populations that give rise to the granule neuronal population of the DG, they found that cilia were affected only in the subgranular zone (SGZ) newborn neurons and not in neurons originating from the dentate neuroepithelium (DNe) [37]. Notably, while astrocytes of the DG in *Fmr1*-KO mice did not exhibit ciliary deficits [37], another study from the same group, showed that cerebellar *Fmr1*-KO Bergmann glia in the Purkinje cell layer of the posterior cerebellum had downregulated Shh signaling and fewer primary cilia after postnatal day 10 [51]. These studies suggest that loss of FMRP may have a cell type- and age-dependent effect on primary cilia, but the mechanism by which FMRP impacts cilia remains unknown.

### 3.2. Tuberous Sclerosis Complex 

Tuberous Sclerosis Complex (TSC) is a developmental multisystem Mendelian disorder that is caused by loss of either the *TSC1* or *TSC2* genes [40,41,52]. The products of these genes, TSC1 and TSC2 proteins, together with the protein TBC1D7, form a heterotrimeric complex that negatively regulates mTOR signaling pathway [40,41,52]. TSC affects multiple organs, including the brain, and patients often present with various neurological and neuropsychiatric conditions including focal malformations called tubers, ASD, intellectual disability, and early onset epilepsy [40,41,52].

Several studies in different models have showed that loss of *Tsc1* or *Tsc2* genes from kidney cells and embryonic fibroblasts leads to impaired ciliary structure [53,54,55]. *Tsc1*-KO or *Tsc2*-KO mouse embryonic fibroblasts (MEFs) were more likely to be ciliated with elongated cilia in comparison to wild-type control MEFs [53]. Notably, a separate study in *Tsc1*-KO or *Tsc2*-KO MEFs observed that while cilia in *Tsc1*-KO MEFs were elongated, cilia in *Tsc2*-KO MEFs were shorter when compared to control [54]. Additionally, loss of *Tsc1* from mouse in the distal convoluted tubules cells led to elongated cilia [55]. Collectively, these studies indicate that TSC1 and TSC2 proteins are important for cilia function, and further studies are necessary to resolve the discrepancies observed in the cilia phenotypes.

A recent study, by Di Nardo et al., explored how loss of *TSC1* and *TSC2* genes affects primary cilia in surgically resected patient brain samples and rodent models of TSC [39]. Loss of either gene in patient samples and in vitro or in vivo rodent models led to a reduction in the number of neuronal primary cilia (Figure 2, Table 1). Notably, the authors showed that shRNA mediated knockdown of *Tsc2* in rat primary hippocampal neurons resulted in loss of primary cilia over time suggesting that the TSC2 protein function and consequent mTOR suppression may be important for cilia stability and maintenance. Furthermore, deciliation could be prevented by knockdown of the heat shock protein 27 (Hsp27), which was upregulated upon loss of *Tsc2* [39]. 

Interestingly, while rapamycin treatment was able to prevent loss of cilia, it was not sufficient to reverse this phenotype [39] suggesting that there is a critical temporal window for mTOR inhibition to be effective in preventing deciliation. Rapamycin and other related compounds (rapalogs) are FDA approved for treatment of multiple manifestations of TSC. While these drugs are effective at treating some neurologic abnormalities, they are not effective in treating TSC-related cognitive deficits [56,57,58]. Of note, these patients were all treated in late childhood or adolescence, which may be ineffective in preventing loss of cilia. Hence, it would be worthwhile to explore whether development of therapeutics aiming to restore or prevent primary cilia deficits could be a more effective therapeutic strategy for cognitive deficits in TSC and potentially other disorders. 

### 3.3. Focal Cortical Dysplasia

Focal cortical dysplasia (FCD) belongs to a group of disorders that are collectively referred to as focal malformations of cortical development (FMCD) [42,43]. FCD is characterized by the presence of hypertrophic “balloon-like” cells, dysplastic neurons with abnormal orientation and processes, disorganized cortical lamination, and gliosis. FCD is one of the most common underlying causes of refractory pediatric epilepsy and intellectual disability [42,43]. 

A recent study by Park et al., examined the role of primary cilia in the pathogenesis of FMCDs caused by somatic mutations in the *mTOR* gene. The authors examined surgically resected tissue from patients and found a reduction in the number and the length of neuronal primary cilia [44] (Figure 2, Table 1). To explore the molecular mechanisms underlying these defects, the authors utilized several models, including in utero electroporated mice that expressed mTOR harboring a somatic mutation identified in FCD patients. Notably, they found that mTOR-related autophagy defects led to accumulation of oral-facial-digital syndrome 1 protein (OFD1) at the centriolar satellites [44], which was previously shown to inhibit ciliogenesis [59]. They showed that *Ofd1* suppression was sufficient to restore primary cilia in mTOR mutant cells. They proposed that OFD1 accumulation and impaired ciliogenesis disrupt Wnt signaling, leading to the cortical dyslamination phenotypes observed in their models [44].

Cortical malformations are also present in multiple developmental disorders, including TSC and phosphatase and tensin homolog (PTEN) hamartoma tumor syndrome (PHTS) [60]. Thus, it would be very interesting to examine whether the mechanism identified in this study is also implicated in the cortical malformation phenotypes in these disorders. 

### 3.4. Cyclin-Dependent Kinase-like 5 Deficiency Disorder

CDKL5 (cyclin-dependent kinase-like 5) Deficiency Disorder (CDD) is a rare genetic disorder characterized by severe neurologic manifestations, including infantile epileptic encephalopathy and cognitive disabilities [46,47,61] and has been also associated with ASD [62,63]. The *CDKL5* gene is an X-linked gene that encodes a serine/threonine kinase, which is highly expressed in the brain and known to be critical for proper dendritic arborization, axonal growth, and synaptic plasticity [64]. 

The importance of CDKL5 in cilia function and structure has been proposed by several studies [65,66,67]. Specifically, it has been shown that CDKL5 localizes to the centrosome, controls ciliary length, and is a key component of ciliogenesis in several systems, including *C. elegans*, *Chlamydomonas*, and proliferating cells lines such as HeLa [65,66,67]. While there are not many studies describing the role of CDKL5 in neurons, one recent study, using in vitro and in vivo rodent models of CDD showed that *Cdkl5* null neurons exhibit increased primary cilia length [45] (Figure 2, Table 1). Even though the relationship between CDKL5 and primary cilia has been explored, the molecular mechanisms underlying this relationship, and the consequences of the neuronal cilia structural changes remain elusive. 

### 3.5. Rett Syndrome 

Rett syndrome (RTT) is a monogenic X-linked rare neurodevelopmental disorder. RTT patients exhibit significant developmental regression and intellectual disability [49]. Mutations in the methyl-CpG-binding protein 2 (*MeCP2*) gene is the leading cause of Rett syndrome [49,68]. MeCP2 is a multifunctional epigenetic regulator that controls the expression of several genes. Notably, MeCP2 recognizes histone methylation marks and can act either as a transcriptional activator or repressor, depending on the presence of various cofactors [69,70,71]. Studies in rodent models explored the effects of MeCP2 loss from the brain and found that this protein is important for energy metabolism and proteostasis [72].

MeCP2 has been shown to localize at the centrosome, affecting the cell cycle and the cytoskeleton stability [73], suggesting that this protein might be important for ciliogenesis. A recent study examined loss of *Mecp2* in several mouse models including primary cortical neuronal and astrocytic cultures as well as fibroblasts from RTT patients. The authors found that loss of *MeCP2* led to fewer and shorter primary cilia and associated reduction in the Shh signaling pathway activity (Figure 2, Table 1). Interestingly, this phenotype could be rescued with a histone deacetylase 6 (HDAC6) inhibitor suggesting that microtubule instability contributes to the primary cilia deficits observed in these models [48]. Notably, while this study provides strong evidence that support a relationship between MeCP2 function and primary cilia, another study showed that of loss of *Mecp2* in mouse retina cells did not affect cilia formation [74]. Taken together, these studies suggest that MeCP2 effect on primary cilia might be cell-type dependent.

## 4. Ciliary Defects as a Convergent Mechanism for Neurologic Phenotypes 

Several of the aforementioned monogenic neurodevelopmental disorders exhibit common neuropsychiatric manifestations including early onset epilepsy, intellectual disability, and ASD. In addition, the majority of these disorders exhibit similar neuronal primary cilia defects, namely fewer and shorter cilia (Table 1). Given the critical role of primary cilia in the brain, it is possible that defects in these organelles contribute to the neuropsychiatric manifestations. It is worth noting however, that primary cilia dysfunction might cause different changes on the molecular level depending on the cell type and the specific genetic perturbation. Hence, while the phenotypic manifestations are shared, different mechanisms could be responsible in each disorder. Therefore, it is important to examine (1) whether and how the genetic perturbations underlying these disorders lead to abnormal cilia structure and function, and (2) if and to what extent, primary cilia dysfunction is involved in this wide range of neurologic abnormalities in each of these disorders. Such studies could open the road for discovery of novel therapeutic targets and development of new treatment strategies.

### Crosstalk between Signaling Pathways in Monogenic Neurodevelopmental Disorders 

Most studies identifying deficits in primary cilia in monogenic neurodevelopmental disorders have not characterized the mechanisms involved. Some of these studies, however, have suggested that alterations in major cellular functions, such as autophagy, are involved [44,75]. It is unclear whether there is a convergent mechanism or independent mechanisms underlying cilia deficits in each of these diseases. While each disorder likely has unique features, several lines of evidence support bidirectional interactions among key components of the molecular cascades involved in these disorders. Interestingly, many potential shared mechanisms center around direct or indirect mTOR dysregulation [76].

Genetic mutations underlying FCD and TSC affect mTOR signaling directly by altering the balance between activation and inhibition [40,43]. However, there are also proposed interactions between the RTT related protein, MeCP2, and mTOR protein. Specifically, aberrant mTOR signaling has been shown in patients with RTT syndrome [77]. In addition, *Mecp2* mutations in mouse models of RTT lead to downregulation of mTOR signaling activity and reduced neuronal size [78], a known phenotype controlled by the mTOR pathway. Interestingly, in *Mecp2* null or heterozygous mice, downregulation of the phosphorylated form of ribosomal protein S6 (p-rpS6), a well-established mTOR target, is detectable prior to the appearance of obvious RTT-related neurologic manifestations [79]. mTOR signaling was also found to be altered in FXS [75,80]. For example, Sharma et al. showed increased mTORC1 activity in the hippocampal region of a mouse model of fragile X [80]. Additionally, Yan et al., showed that mTOR-dependent decreased autophagy is responsible for several of the phenotypes observed in *Fmr1*-KO mice, including spine and synaptic plasticity defects as well as impaired cognition [75]. MeCP2 was also shown to interact with the FMRP. Specifically, a study showed reciprocal regulation between the expression levels of these two proteins both in vitro and in vivo [81].

CDKL5 has also been shown to affect the mTOR pathway [82,83,84]. Studies in *Cdkl5* mutant mouse models reveal downregulation of Akt and mTORC1 activity, hence disruption of the Akt/mTOR signaling cascade [83,84]. Notably, one of these studies showed that by boosting phosphorylation of GSK-3b, an Akt downstream target, in *Cdkl5* null neuronal precursor cells, several developmental alterations including neuronal survival and maturation were rescued [84]. Another study examined how loss of Cdkl5 affected the mTOR signaling cascade by examining components of the mTOR pathway in different neuronal types. The authors examined cortical excitatory and inhibitory neurons, as well as striatal inhibitory neurons, and observed differential perturbation of the mTOR signaling cascade, suggesting that Cdkl5 affects mTOR in a cell type-dependent manner [82].

The mTOR dysregulation seen in FCD, TSC, RTT, FXS and CDD is noteworthy given that several lines of evidence propose that that mTOR and primary cilia regulate each other [85]. Specifically, primary cilia inhibit mTORC1 activity via several proposed mechanisms involving proteins such as Lkb1, Folliculin, AMPK and polycystin-1 [86,87,88]. Reciprocally, several studies have shown that mTORC1 activity affects cilia formation and length [44,89,90]. 

Outside of mTOR, MeCP2 and CDKL5 have been shown to interact in various systems. Specifically, it has been shown that MeCP2 can be phosphorylated in a Cdkl5-dependent manner [91,92] and that Cdkl5 is a MeCP2-repressed target gene in the rat brain [93]. In addition, patient stem cells that express mutated MeCP2 or CDKL5 exhibit common phenotypes such as upregulation of glutamate D1 receptor (GluD1) [94].

Taken together these data suggest that there is some crosstalk between components of these signaling pathways and support the hypothesis of a convergent mechanism that could act independently or synergistically with other mechanisms to underlie cilia defects. One of the most intriguing signaling cascades that appears dysregulated in all these disorders is the Akt/mTOR signaling pathway. In FCD, TSC and FXS models Akt/mTOR appears upregulated and the number of primary neuronal cilia is reduced [37,39,40,43,44,75,80]. Additionally, in FCD and FXS, remaining primary cilia are also shorter in length [37,44]. On the other hand, a few studies have shown that in CDD, Akt/mTOR is downregulated and primary neuronal cilia length is increased [45,82,83,84]. Taken together these data suggest that Akt/mTOR activity can bidirectionally affect the number and length of primary neuronal cilia. However, RTT syndrome appears to be an exception to this Akt/mTOR activity–cilia phenotype pattern as mTOR in RTT is downregulated and primary cilia are reduced both in number and length [48,78,79]. One explanation could be that different mechanisms in each genetic perturbations could underlie and/or contribute to the primary cilia phenotypes. Further studies are warranted to elucidate the molecular mechanisms leading to impaired ciliation in these disorders. 

## 5. Impact of Primary Cilia Loss in Neurons and Neuronal Networks

While primary cilia defects have been identified in the neurodevelopmental disorders discussed above, the extent of their contribution to the neurologic abnormalities remains elusive. Neuronal primary cilia regulate a plethora of signaling cascades and extensive studies have shown that their proper function is crucial both in the developing and the adult brain [34,35,95]. These studies, a few of which will be discussed here, can shed light into the potential contributions of primary cilia into brain-wide disease phenotypes such as structural deficits, seizures, and cognitive disabilities. (For a more comprehensive list of studies focusing on mouse models see Table 2 and Reviews [96,97,98]).

Numerous studies have examined the roles of primary cilia in the developing brain [32], since these organelles regulate signaling pathways essential for brain patterning, neuronal migration and differentiation (reviewed elsewhere) [95,116,117]. These studies have provided a significant framework to explore the contribution of cilia in disease phenotypes. Interestingly, impaired neuronal migration is observed in some of the monogenic neurodevelopmental disorders discussed above such as in TSC, RTT and FMCDs [40,43,118]. Notably, one study examining models of FCD, showed that aberrant Wnt signaling due to cilia loss was the underlying cause for impaired neuronal migration and cortical lamination deficits [44], supporting the key role of primary cilia in proper brain development. 

Abnormal neuronal and network activity is involved in seizures and cognitive deficits such as ASD which are common phenotypes in several neurodevelopmental disorders [119,120]. Recent studies have revealed that primary cilia are important for proper neuronal structure, function, and network connectivity [108,110,114,121], suggesting that cilia deficits might play a role in these neurologic manifestations. Specifically, a study by Kumamoto at al. showed that primary cilia are essential for the integration of hippocampal neurons into existing neuronal circuits [108]. In this study the authors manipulated primary cilia in mouse adult-born neurons and showed that newborn dentate granule cells (DGCs) lacking primary cilia, exhibit glutamatergic synapse formation defects and dendritic refinement deficits. The authors also noted that loss of primary cilia enhanced Wnt/β-catenin signaling activity and expression of a constitutively active form of β-catenin in newborn DGCs was sufficient to recapitulate the dendritic defects [108]. Another study by Rhee et al., explored the effects of primary cilia depletion from mouse mature DGCs and found hippocampus-dependent memory and synaptic plasticity defects [110]. Specifically, the authors showed that these mice exhibited impairment in contextual fear and spatial recognition memory. Further examination of brain slice preparations revealed increased long-term potentiation (LTP) in the CA3 region, which could potentially account for the observed behavioral changes [110]. Bowie et al. deleted the *tau tubulin kinase 2* (*Ttbk2)* gene which encodes for an essential regulator of ciliogenesis in young adult mice, using a tamoxifen inducible line and examined the effects of cilia loss in the cerebellum [114]. When they examined Purkinje cells, the authors found significant defects including altered intracellular Ca^2+^ concentrations, loss of excitatory synapses from climbing fibers, and Purkinje cell death [114]. This study signifies the importance of cilia in the maintenance of connectivity between neurons, as well as neuronal survival [114]. Interestingly, another study from Tereshko et al., showed that acute disruption of ciliary signaling in rat cortical cultured neurons lead to strengthening of glutamatergic synapses and increased spontaneous firing, while there was no effect on dendritic morphology, passive neuronal properties, or intrinsic excitability [121]. 

Besides their role as sensory “antennae”, neuronal primary cilia were recently showed to be sites of synaptic contacts [122]. The axo-ciliary synapse was first showed by Sheu et al., using enhanced focused ion beam-scanning electron microscopy [122]. The authors discovered functional synapses between brainstem serotonergic axons and primary cilia from CA1 hippocampal neurons that express serotonin (5-hydroxytryptamine, 5-HT) receptor type 6 (5-HTR6) [122]. They also found that stimulation of this type of receptor is linked to nuclear actin modifications in post-mitotic and post-migratory CA1 neurons [122]. Taken together these studies highlight the importance of primary cilia in proper neuronal morphology, function, and connectivity.

### Primary Cilia Signaling Pathways in Brain Pathology

Several models of the neurodevelopmental disorders discussed in this review show that ablation of primary cilia occurs only in a minority of neurons, sparing most neuronal primary cilia. For example, Di Nardo et al., showed that in the hippocampus of *Tsc2*-KO mice there is on average only a ~20% decrease in the number of ciliated neurons within the CA1 region in comparison to WT mice [39]. A similar magnitude of decrease in ciliation was observed in an *Fmr1*-KO mouse model where the authors noted that only a subset of DG neurons had lost their cilia [37]. Surprisingly, most neurons in these models have seemingly structurally intact cilia. However, it is still unknown whether the remaining neuronal cilia retain normal function, and if not, whether their impaired function contributes to brain pathology. 

Primary neuronal cilia in the brain express components of numerous signaling pathways, with several receptors being localized either primarily or exclusively in the primary cilium [21,22,123]. In order to elucidate the roles of specific pathways some studies selectively manipulated receptors and key components affecting the activity of these pathways while retaining the cilium structure intact. For example, Einstein et al., examined the effects of somatostatin receptor type 3 (SSTR3) loss in mice [103]. SSTR3 is a GPCR, located exclusively in primary neuronal cilia in the brain [124,125]. The authors examined *Sstr3*-KO mice and found that there is no apparent disruption of cilia structure nor changes in the number of primary cilia. Interestingly, this study revealed that SSTR3 signaling is critical for hippocampal synaptic plasticity and object recognition memory [103]. A more recent study by Tereshko et al., further showed that SSTR3 almost exclusively localizes to primary cilia of excitatory neurons in the cerebral cortex in rodent models and modulates excitatory synaptic properties [121]. Melanin concentrating hormone receptor 1 (MCHR1) is another GPCR abundantly expressed in neuronal primary cilia within several brain structures including cerebral cortex, hippocampus, and amygdala [126]. Adamantidis et al. showed that MCHR1 knock out mice exhibited reduced NMDA receptor 1 subunit in the CA1 region and deficits in learning and memory [99]. Another study exploring the role of 5-HTR6 signaling reported that *5-Htr6*-KO mice exhibited cognitive impairments and had altered gene expression, impaired morphology and physiology in hippocampal neurons [115]. 

Type 3 adenylyl cyclase (AC3) belongs to the cAMP signal transduction pathway predominantly localizes to primary cilia throughout the brain [127]. Several studies examining AC3 knock out mice have reported phenotypes such as obesity, major depression, and learning and memory deficits [106,127]. Small GTPase ADP-ribosylation factors (ARF), are essential for membrane trafficking [128]. Arl13b, a small GTPase that belongs to the ARF family, is highly enriched in primary cilia. Loss of Arl13b from mouse cortical progenitor cells leads to abnormal neuronal placement in the developing cerebral cortex [109]. Another study examined how conditional deletion of Arl13b from cortical projection neurons and interneurons affects their migration. Notably the authors found that Arl13b is specifically required for proper interneuron cortical migration but not for cortical projection neurons [107]. Another study found that loss of Arl13b from postnatal interneurons in the striatum caused reduction of dendritic and axonal complexity, synaptic connectivity deficits, and altered Ca^2+^ signaling and ciliary localization of GPCRs [113]. Interestingly, chemogenetic activation of GPCR signaling or expression of Sstr3 in the Arl13b deficient interneurons was sufficient to rescue morphology and connectivity abnormalities [113]. Changes in interneuron migration or morphology can result in changes in brain’s network activity and connectivity. Such alterations could lead to imbalanced excitation/inhibition (E/I) ratio which has been implicated in several neuropsychiatric conditions including epilepsy and autism [120]. 

Overall, these studies suggest that aberrant ciliary signaling can be sufficient to drive neurologic abnormalities. Therefore, it is important to develop tools to measure functional changes of primary cilia that appear structurally intact in neurodevelopmental and other brain disorders. 

## 6. Conclusions and Future Directions 

The field of neuronal primary cilia has been gaining significant attention in recent years. These organelles, which were once thought to be vestigial structures, now appear to be key players not only for proper brain development but also for proper function of the adult brain, and they are implicated in a wide range of neurologic and neuropsychiatric disorders [32,34,35]. However, the functions of neuronal primary cilia remain enigmatic due to the substantial complexity and breadth of actions they exhibit which can vary significantly based on the cell-type, age, and brain region. The implications of primary neuronal cilia in neurologic and neuropsychiatric presentations, however, have prompted efforts to generate tools and assays to characterize their function. 

Major efforts are currently focused on dissecting the proteomic composition of primary cilia in different cell types [129,130,131,132]. Such studies have already revealed novel key components of cilia signaling pathways [133]. Identifying proteins unique to cilia subtypes will pave the way for our understanding of how primary cilia contents change to support the function of specific neuronal and glial cell types. Furthermore, development of tools that will enable the study of cilia function and structure are also currently in progress. A group recently developed a novel approach to study ciliary signaling and function using a nanobody-based targeting approach combined with optogenetics tools and biosensors [134]. Moreover, another exciting area currently under development is the functional and structural assessment of primary cilia via application of new imaging methods [122,135]. Imaging techniques including expansion microscopy [135] and enhanced focused ion beam-scanning electron microscopy [122] have already uncovered novel roles for neuronal cilia such as the fact that they can be sites of synaptic contact [122]. 

In summary, while the primary cilia field is still at its infancy, there have been increasing efforts to understand the role of these organelles in the healthy and diseased brain. Advancing our knowledge in neuronal primary cilia biology and shedding light on the contributions of these organelles to brain pathologies will potentially facilitate the development of novel therapeutic targets and treatment strategies for neurologic abnormalities.

## Figures and Tables

**Table 1:** Primary cilia phenotypes in neurodevelopmental disorders discussed in this review.

Mutated Gene	Disease	Key Neurologic Features *	Experimental Systems ^+^	Neuronal PC Phenotypes ^+^	Ref
*FMR1*	Fragile X Syndrome (FXS)	ASD, ID, seizures, ADHD, neuroanatomical abnormalities, i.e., larger volume of lateral ventricles	*Fmr1*-KO mice	↓ Number ↓ Length	[36,37,38]
*TSC1/2*	Tuberous Sclerosis Complex (TSC)	Tubers, SENs, SEGAs, epilepsy, disorganized WM, ID, ASD, ADHD	*SynCre*; *Tsc1*^c/c^ mice TSC patient tissue *Tsc2*-KD Primary rat hippocampal neurons ^#^ SynCre; *Tsc2* ^k/c or c/c^ TSC patient tissue	↓ Number	[39,40,41]
*mTOR*	Focal Cortical Dysplasia (FCD)	Epilepsy, ID, ASD, altered cortical architecture	FMCD patient Samples Genome edited mTOR p.Cys1483Tyr NIH/3T3 cell lines In utero electroporated mice-p.Cys1483Tyr-IRES-EGFP	↓ Number ↓ Length	[42,43,44]
*CDKL5*	CDKL5 Deficiency Disorder (CDD)	Infantile spasms, ASD, epilepsy, ID	*Cdkl5*-KD primary rat hippocampal neurons *Cdkl5*-KO mice	↑ Length	[45,46,47]
*MeCP2*	Rett Syndrome (RTT)	ID, ASD, seizures	Mouse embryonic fibroblasts Primary mouse cortical cultures *Mecp2*-KO mice RTT patient fibroblasts	↓ Number ↓ Length	[48,49]

^*^ The primary neurologic features of these disorders are listed based on the references noted, as well as in the OMIM database and the NIH Genetic and Rare Diseases Information Center. Other neurologic phenotypes may be present in these disorders and not all patients may present the ones listed here. ^+^ Listed here are the primary cilia phenotypes and the experimental systems from the studies discussed in this review. ^#^ k allele is full KO while c allele is a conditional mutation leading to ~7% expression of Tsc2 protein. (ASD)Autism spectrum disorder, (ID) Intellectual disability, (ADHD) Attention deficit hyperactivity disorder, (SENs) subependymal nodules, (SEGAs) subependymal giant cell astrocytomas, (WM) White matter, (KD) Knockdown, (FMCD) Focal malformations of cortical development. Arrows indicate either reduction or increase.

**Table 2:** Ciliary, neurological, and behavioral phenotypes observed in primary cilia deficit mouse models.

Mouse Model	Primary Cilia Phenotypes *	Neurological Phenotypes ^+^	Behavioral Phenotypes ^+^	Ref
*Mchr1^Neo/Neo^*	n/a	↓ Nmdar1 mRNA in CA1	Impaired learning and memory	[99]
*Dnchc2* mutant	Structurally impaired in neuroectoderm	Dorsoventral patterning defects Randomized left-right axis	n/a	[100]
*hGFAP-Cre*; *Kif3a^fl/fl^*	Loss from granule neuron precursors in DG	Lack of postnatal neurogenesis DG hypotrophy Defective Shh in DG	n/a	[26]
*Emx1-Cre*; *Shh^fl/-^* *Emx1-Cre*; *Smo^fl/-^*	n/a	Smaller DT ↓ neural progenitor/stem cell proliferation ↑ cell death	n/a	[101]
*Cobblestone* (hypomorphic *Ift88*)	Normal morphology in ventricles	DT disorganization ↑ canonical Wnt in neocortex and caudal forebrain	n/a	[102]
*Sst3* knockout	Normal in CA1	Disrupted cAMP mediated LTP	Impaired recognition memory	[103]
*Ift20*^*fl*/*fl*^::*mGFAP-Cre*	Loss from radial neural stem cells in the SGZ	↓ hippocampal amplifying progenitors ↓ hippocampal neurogenesis	Delay in learning and enhanced cue-based fear responses	[104]
* Ftm * mutant	Loss from telencephalic neuroepithelial cells	Expanded subpallium in anterior telencephalon Ectopic OB	n/a	[105]
*Ac3* mutant	Structurally intact in hippocampus	n/a	Impaired learning and memory	[106]
*Arl13b^fl/fl^*; *Nex-Cre*	n/a	↓ axonal bundles in the IC	n/a	[107]
*Arl13b^fl/fl^*; *Dlx5/6-CIE*	Shorter on interneurons	Disrupted interneuron migration, placement, and branching in cerebral cortex	n/a	[107]
Inducible dominant negative *Kif3a* expressed in the hilus region of DG of adult mice	Loss from newborn DGCs	Defects in dendritic refinement and synapse formation in newborn DGCs	n/a	[108]
*Arl13b^hnn/hnn^* (null allele)	Shorter with disrupted morphological plasticity on radial progenitors in cortex	Reversal of apical-basal polarity of radial progenitors in cerebral wall Disrupted cortical lamination	n/a	[109]
*Ift20^fl/fl^* AAV-CaMKII-eGFP-Cre injected in DG of adult mice	Loss from majority of GFP+ cells in DG	↑ LTP in MF-CA3 synapses	Impaired memory	[110]
*Cobblestone* (hypomorphic *Ift8*8)	Normal morphology in VZ of ventral midbrain	↓ mDA neurons Disrupted Shh signaling	n/a	[111]
*Nestin-Kif3a^fl/fl^*	Loss from cortical ventricular surface and somatosensory cortex	Enlarged LVs Enlargement of RGCs apical domains ↑ number of BPs ↑ cortical thickness	n/a	[112]
*Nkx2.1Cre*; *Arl13b^fl/fl^*	Defective intraciliary Ca^2+^ signaling in striatal interneurons	Neuronal morphology defects Disrupted synaptic connectivity	n/a	[113]
*Ttbk2^fl/fl^*; *Ubc-Cre-ERT2*^+^ (Tamoxifen on P21)	Loss from cerebellum, brainstem, hippocampus, and cortex	Loss of VGLUT2^+^ synapses on Purkinje cells Altered intracellular Ca^2+^ in Purkinje cells ↓ number of Purkinje cells	Locomotor deficiencies	[114]
*5-ht6r* mutant	Normal morphology in hippocampus	Altered Shh ↓ cAMP Altered neuronal morphology and excitability	Anxiety and cognitive impairments	[115]

This table contains information from studies which investigated primary cilia deficits in mouse models. A subset of these studies is discussed in the review. Arrows indicate either reduction or increase. * Listed are the primary cilia phenotypes within the brain regions investigated in the referenced study. ^+^ Listed are the key neurological and behavioral phenotypes reported in the reference study. Some studies might have additional phenotypes not listed in this table. (Nmdar1) N-methyl-D-aspartate receptor subunit 1, (DG) Dentate gyrus, (Shh) Sonic Hedgehog, (DT) Dorsal telencephalon, (LTP) Long-term potentiation, (SGZ) Subgranular zone, (OB) Olfactory bulb, (IC) Internal capsule, (DGCs) Dentate granule cells, (MF) Mossy fibers, (RGCs) Radial glia cells, (VZ) Ventricular zone, (mDA) Midbrain dopaminergic, (BP) Basal progenitors, (LVs) lateral ventricles, (VGLUT2) vesicular-glutamate transporter 2.

## Data Availability

Not applicable.

## Abbreviations

AC3	Type 3 adenyl cyclase
ARF	ADP-ribosylation factors
ASD	Autism spectrum disorder
CDD	CDKL5 deficiency disorder
CDKL5	Cyclin-dependent kinase-like 5
CNS	Central nervous system
DG	Dentate gyrus
DGC	Dentate granule cell
DNe	Dentate neuroepithelium
E/I	Excitation/inhibition
FCD	Focal cortical dysplasia
FGF	Fibroblast growth factor
FMCD	Focal malformations of cortical development
FMRP	Fragile X messenger ribonucleoprotein
FXS	Fragile X syndrome
GluD1	Glutamate D1 receptor
GPCR	G-protein-coupled receptor
HDAC6	Histone deacetylase 6
Hsp27	Heat shock protein 27
ID	Intellectual disability
IFT	Intraflagellar transport
KD	Knockdown
KO	Knockout
LTP	Long term potentiation
MCHR1	Melanin concentrating hormone receptor 1
MeCP2	Methyl-CpG-binding protein 2
MEF	Mouse embryonic fibroblast
mTOR	Mechanistic target of rapamycin
OFD1	Oral-facial-digital syndrome 1 protein
PDGFα	Platelet-derived growth factor α
PHTS	PTEN hamartoma tumor syndrome
PKD	Polycystic kidney disease
p-rpS6	Phosphorylated ribosomal protein S6
PTEN	Phosphatase and tensin homolog
RTT	Rett syndrome
Shh	Sonic hedgehog
SGZ	Subgranular zone
SSTR3	Somatostatin receptor type 3
TEM	Transmission electron microscopy
TGF-β	Transforming growth factor β
TSC	Tuberous sclerosis complex
Ttbk2	Tau tubulin kinase 2
5-HT	5-hydroxytryptamine
5-HTR6	5-HT receptor type 6
